# Mapping Ethical Blind Spots in the Use of New Technologies to Support Elder Care

**DOI:** 10.1007/s11673-025-10502-y

**Published:** 2025-12-23

**Authors:** Tenzin Wangmo, Yi Jiao Angelina Tian, Emilian Mihailov, Lester D. Geneviève

**Affiliations:** 1 Institute for Biomedical Ethics, University of Basel, Bernoullistrasse 28, 4056 Basel, Switzerland; 2 Research Center for Applied Ethics, Faculty of Philosophy, University of Bucharest, 204 Splaiul Independenţei, Sector 6, 060024 Bucharest, Romania; 3 Faculty of Medicine, Laval University, 1050 Av. de La Médecine, Québec City, Quebec G1V 0A6 Canada

**Keywords:** Ethics, Technology, Smart technologies, Elder care, Qualitative study

## Abstract

This paper maps the ethical concerns involved in using new technologies in the care of older persons by type of participant group and technology used. The study presents data from sixty-seven participants in total from three stakeholder groups (older persons, professional caregivers, and family caregivers) who relayed their opinions on three types of technologies: wearables, ambient sensors, and social assistive robots. The interview data collected was analysed using content analysis. Participants raised ethical concerns about user control, stigma, over-reliance on technology, isolation, deception, privacy breaches, and the replacement of human care. However, these concerns were not uniformly emphasized across participant groups, and not all concerns applied to each of the three technologies. Our findings revealed a reduced ethical sensitivity and preparedness for risk in the case of certain technologies. These ethical blind spots evident in our results pertained to privacy, stigma, and deception. With technologies continuously developing, such blind spots are a cause for concern.

## Introduction

The rising interest in gerontechnologies in the last decade is visible in the number of literature reviews available on new technologies—such as intelligent assistive technologies, smart homes, and social robots—illuminating their availability, benefits, and challenges (Demiris and Hensel 2008; Ienca et al. 2017; Liu et al. 2016; Majumder et al. 2017; Marikyan, Papagiannidis, and Alamanos 2019; Tian, Felber, et al. 2024; Wilson, Hargreaves, and Hauxwell-Baldwin 2015). These technologies can either be embedded within the home space or be used as a single technology to support, for example, older users with activities of daily living, monitor their health and safety, and assist with cognitive and physical needs. In doing so, they enable older adults to age independently at home and consequently reduce caregiving costs and burdens (Liu et al. 2016; Majumder et al. 2017; Turjamaa, Pehkonen, and Kangasniemi 2019). The aging of the global population, particularly in high-income countries, is creating an imbalance between the increasing healthcare needs of older adults and a declining number of both formal and informal caregivers. This trend highlights, to some extent, the inevitability of having to use technology to care for older adults, both at home and in institutional settings (Huang and Oteng 2023; Vermeer, Higgs, and Charlesworth 2019).

Many ethical and social concerns have been raised in the hope of ensuring the appropriate introduction of these technologies in elder care (Novitzky et al. 2015; Sharkey and Sharkey 2012; Zwijsen, Niemeijer, and Hertogh 2011). An ethnographic study of participants’ use of digital technology at home revealed that users wish to have control over the technology and the flow of information (Burrows, Coyle, and Gooberman-Hill 2018). Sorell and Draper (2014) defend the significance of respecting the autonomy of the user when robots are deployed as carers. Other concerns discussed include user centeredness, obtrusiveness, privacy, data safety, deception, stigma, overdependence on technology, and the potential replacement of human care (Bowes, Dawson, and Bell 2012; Coghlan et al. 2021; Demiris et al. 2004; Felzmann et al. 2015; Ienca et al. 2018; McLean 2011; Mittelstadt 2017; Novitzky et al. 2015; Sharkey and Sharkey 2012).

A deeper understanding of how the use of such technologies in elder care differs for the older person and their caregivers is necessary to learn the attribution of these ethical values across different technologies. Yet, many studies provide only a partial view of the situation by identifying ethical concerns raised from a narrow range of perspectives—generally, a small sample of older adults, or only caregivers or tech developers—, and oftentimes by focusing on one specific technology, like robots (Birchley et al. 2017; Coghlan et al. 2021; Ienca et al. 2018; Vandemeulebroucke et al. 2020).

In contrast to the available studies, our aim is to map ethically relevant topics relative to types of new technologies and to participant groups. First, this mapping will allow scholars, older persons who may use these technologies (i.e. end-users), caregivers, and tech developers to be cognizant of why and how a specific technology is used and who in the end benefits from it, whether directly and indirectly. The needs and interests of different stakeholders may not necessarily align. Second, based on our findings, we want to add to the existing literature by examining its ethical blind spots; that is, patterns of reduced ethical sensitivity and ethical preparedness for the risks that certain types of technology may incur. This is important in light of the speed at which technology development is taking place.

## Method

This paper stems from a mixed-methods research project in which we aimed to capture older persons’ and their caregivers’ existing knowledge, attitudes, and conditions for accepting the use of new technologies in elder care. The project involved systematic literature reviews (Felber et al. 2023; Tian et al. 2024), theoretical analysis (e.g. Felber et al. 2025), qualitative interviews (e.g. Tian, Duong, et al., 2024; Wangmo et al., 2024; Mihailov and Wangmo, 2025), and a representative survey of adults in Switzerland (Wangmo et al. 2025). For the qualitative component, we used an exploratory qualitative study design to elicit the perspectives of three different stakeholder groups. The empirical part of the project was evaluated by the Ethics Commission of Northwest and Central Switzerland. Using empirical research to identify ethical issues in practice illustrates a bottom-up approach, in which existing ethical issues in practice are identified and studied. Empirical research makes us open to whatever issues exist and new ones that are found in addition to available scholarly discussions.

An interview guide was developed and pilot-tested for the project with three sections. The first section consisted of general questions with a view to understanding the participants and their care needs (or care provision, in the case of caregivers). The second section consisted of specific questions for each technology type included in the study: emergency alarm bracelets, wearable sensors, ambient (visual) sensors, social assistive robots (Pepper), pet-like robots (PARO), and virtual reality glasses. The interviewers presented participants with pictures of each technology. For Pepper, participants watched a video clip from YouTube.^1^ Other studies have also used videos and photos instead of real life interaction with technologies to elicit opinions (Kühne et al. 2022), indicating that this method is acceptable in gathering information for similar research purposes (Roesler, Manzey, and Onnasch 2021). The use of pictures and video clips were intended to ensure that participants were relaying their opinions on the technology under study and were not misunderstanding the questions to concern another, irrelevant device. This was important because we did not screen whether prospective participants were already using such technology for care purposes or had a certain level of technical knowledge as part of our recruitment criteria. The different technologies were selected because they represent new technologies that are becoming more ubiquitous (wearables on the body), those that are available and can be repurposed for elder care (sensors at home and cameras), those that are used in geriatric settings (PARO), and finally those which, despite being somewhat futuristic, represent probable candidates for adoption in such settings (Pepper). For each technology introduced, questions eliciting the participants’ impressions and experiences with the technology, the benefits they see in using them, and concerns that they associate with such use were included. Regarding concerns, we probed them to think about financial, social, ethical, and other issues for each technology introduced. The final section asked them to compare the technologies and sought their general recommendations for the use of such technologies in elder care. The interview guide was adapted according to their roles in care, such as their perceptions on whether the technology was used primarily for themselves (as an older participant), or for their care recipients (as a formal caregiver). The interview guide was translated into German and French and back-translated to ensure accuracy.

Purposive and snowball sampling were used to recruit study participants. We advertised our study on social media channels, proactively contacted organizations serving older persons and their care providers, home care providers, nursing homes, assisted living facilities, and Meals-on-Wheels. All participants who contacted us after seeing the advertisements were referred by contacts at the above-mentioned institutions or were referred by prior participants received an information document before the interview date detailing the purpose, procedure, and anonymization measures. The interviewers informed them that withdrawal of their participation was possible at any time and that data collected until that point would be deleted if they chose to do so. The interview commenced after they provided written informed consent to participate in the study.

Two female Swiss interviewers carried out the recruitment of study participants and the actual interviews. At the time of the interviews, one was completing her doctoral education in biomedical ethics and the other was supporting the project as part of her master’s degree in medicine. They received training in qualitative methodology. They interviewed sixty-seven participants for the study, including twenty-seven older persons (of which fifteen were older females), seventeen family carers (thirteen female carers), and twenty-three professional care providers (nineteen female professionals). The older persons were either living independently at home or in nursing homes/assisted living facilities. Family carers were looking after either an older person with dementia or with other care needs. The professional carers were nurses, nurse-aids, home nursing care providers, and those working in assisted living facilities. The average age of the older persons was 87.4 years (73–99 years), that of family carers was 57.1 years (26–83 years), and professional care providers was 45.2 years (31–63 years). Study participants chose the venue for the interviews (mostly their homes) and the interviews lasted on average 96 min (46–189 min). The interviews took place between September 2021 and October 2022. No repeat interviews were done, and transcripts were not returned to participants for checking.

The two interviewers transcribed all interviews verbatim from Swiss German (dialect) into High German. For this paper, using inductive coding (a bottom-up approach), the authors extracted all ethically relevant data from the discussions with the sixty-seven participants concerning wearables, sensor technologies used at home, and the robot Pepper. Findings for the robot PARO and virtual reality were not included here, as they have been written separately elsewhere (Felber, Mihailov, and Wangmo 2025; Wangmo et al. 2024). This ethically relevant dataset—which was initially inductively coded by two project team members in MAXQDA, and includes codes such as “autonomy,” “privacy,” “data sharing,” “cost,” “warmth/softness,” “benefits,” and “general ethical issues”—was then extracted into an Excel document. The first author read, recoded, and sorted these segments into relevant themes and sub-themes, with emphasis on type of technology and participant group, using content analysis (Hsieh and Shannon 2005; Mayring 2000; Schreier 2014). This double coding procedure ensured that the coding process was rigorous. The first author then wrote a detailed draft of all the ethically relevant issues evident in the data and mapped them per study participant group as well as by technology type. The findings were presented and discussed with the co-authors together, and the content presented in the results section was agreed upon. To simplify the results, we combined ambient and visual sensors. We also included some exemplary quotes from the participants to substantiate our interpretation, which we translated from German to English and grammatically corrected to ensure comprehension.

## Results

### Decision-Making

**Fig. 1:**
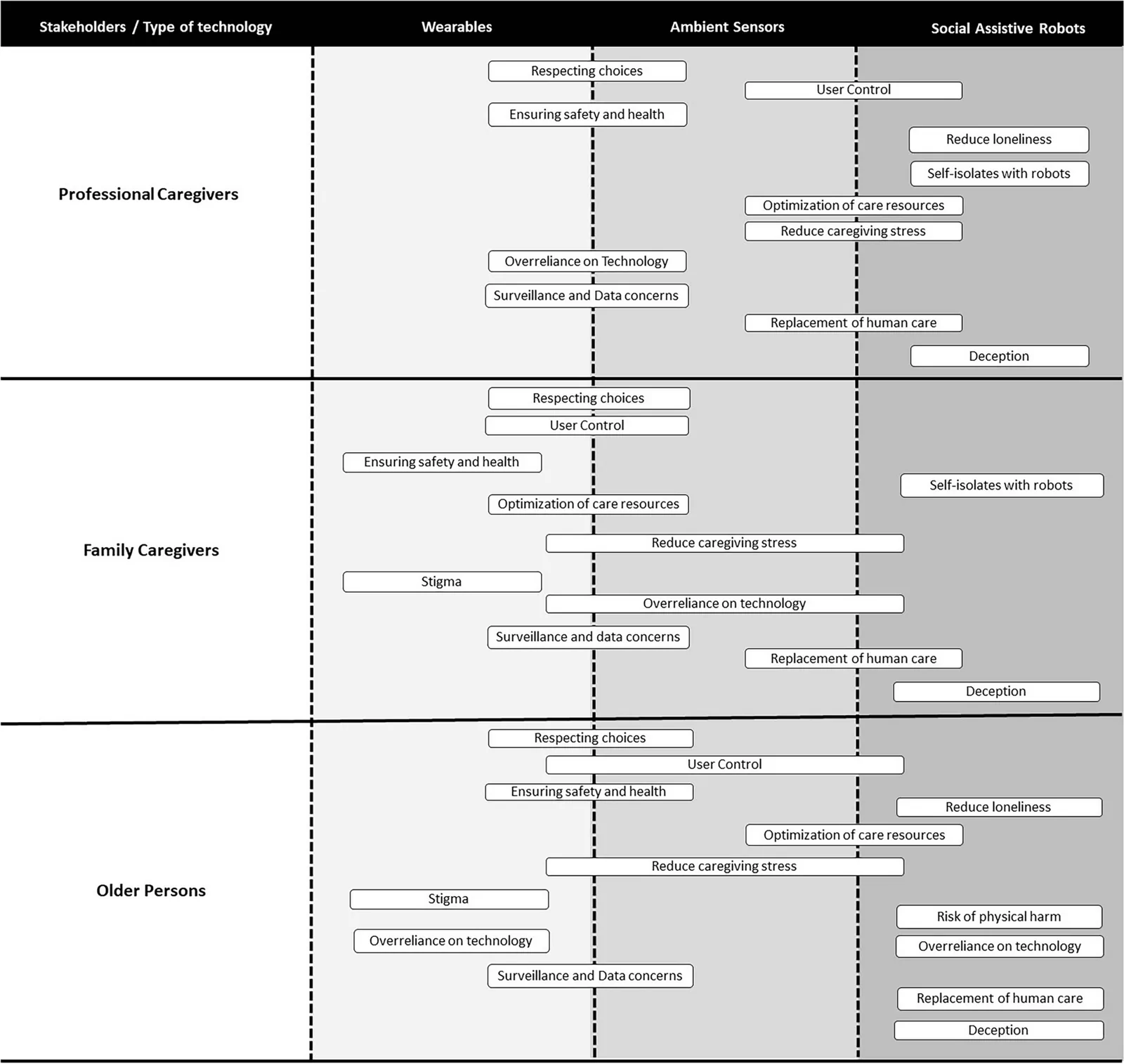
Ethical deliberations in the use of new technologies to care for older persons

All three participant-groups underlined the importance of **respecting choices** made by the end-user for the wearables and the monitoring technologies inquired in this study (Fig. 1). Family caregivers said that the elder user can choose the watch if they wish to, or they can decide against it. In cases of indecision, some of the family caregivers expressed their hopes that the elder parent will be reasonable and would agree to use the technology, since it has a supporting function. For those with dementia who are deemed incompetent to make a legal decision, all three participant groups reported that **proxy decisions** could be made by the healthcare team and/or the family after explaining the purpose of the technology and its utility to the older person.

*Professional caregiver 1*: The most important thing for me in such matters is the consent of the monitored person. So, if I know there is a camera in my kitchen and living room, that is something different from when I find out afterward that someone has been watching me … Or if a nursing home is equipped like this [with sensors and cameras] in a whole building, for example, also in the hallways and the elevator, then there should be transparency. If someone is interested in the nursing home, they should know that … [that] it is a measure by the facility to reduce falls and the time residents spend lying down, just like that.

The notion of **user control** of the technology was evident for all three technologies (see Fig. 1). In the case of the robot Pepper, discussions revolved around the older user being able to set boundaries for what the robots can do and cannot do in order for the user to be safe. Likewise, for wearables, family caregiver and older person participant groups noted that users should decide whether and when to have the smartwatch’s feedback and where the cameras should be installed so that every facet of an individual life is not captured. Finally, related to the sensors at home, family caregivers stated that there is a greater risk of not being in control because, unlike wearables and robots, one cannot easily put sensors away and since video surveillance and ambient sensors run automatically throughout the day.

*Older person 4:* Yes. Otherwise, if he [Pepper] were to become independent, it would be disgusting if he taught himself. You just have to show him the boundaries, “You’re allowed to do that and there you have to …,” when he then says where it is okay …

*Family caregiver 8*: If you have video surveillance, you cannot turn it off, you have it, the sensors are always there, the clock runs 24 hours... so of course it is easy from that point of view, you can put one away [the robot] and the other [sensors] always runs. That is probably why the thresholds here (monitoring) are higher than on the other side.

### Benefits of Using New Technologies for the Older End-user

#### Ensuring Safety and Health

For each technology, the three study participant groups noted that there were certain benefits to the older user. Sensor technologies on the wearables, as well as those in the home, could send alerts in emergency situations, thereby ensuring the **safety of the older person**. The sensor technologies also collect data on their users, which might allow caregivers to detect risks of falls or equip the users with information to **improve or manage their current health conditions**, such as seizures. For instance, one older participant said: “Of course that [wearable] can be positive. I see it from the perspective, when I am no longer able to get help, the help is automated” (Older person 3). Related to health maintenance, the three participant groups felt that the reminder functions were also important for planning and self-management purposes by repeatedly supporting older persons with their medication, nutritional or daily fluid intake, and their physical activity needs (Older person 1: “Yes, I would find that very beneficial for older people because they often don’t drink enough”). This benefit for the safety and health management of the user did not come up for the robotic technology (Fig. 1).

#### Ameliorating Loneliness

Professional caregivers and older study participants saw the robot, Pepper, as a tool that could help older adults **reduce loneliness,** as it could support them in activities such as reading a book, having a conversation, or connecting with relatives who do not reside in the same place (Fig. 1). Pepper was also mentioned as a source of entertainment for older persons with dementia. For instance, one participant said: “You can use it as entertainment, so you can really do a lot of things. And we saw that too, that is, we also saw that [how it was used] for persons with dementia” (Older person 12). However, participants from the caregiver groups stressed the fear that engagement with robots could result in older persons **self-isolating**, by forming human–robot interpersonal relationships at the expense of others.

*Family caregiver 9:* I also see the disadvantage with these technologies, especially with robots, isolation. People go out less because they think, yes, this technical object is enough for me, which means that interpersonal relationships are lost or suffer as a result. That is what loneliness is.... Exactly.

*Professional caregiver 5:* Yes, I would find it strange as well. The relational aspect worries me or raises concerns, where people might suddenly feel they could form a relationship. And, it is not a real relationship. Or rather, that’s not quite right either; it is a relationship, but... a relationship at the push of a button that you can turn off. Maybe it would also prevent them from pursuing other relationships.

### Benefits of New Technology for the Caregivers

#### Optimize Care Resources

Professional caregivers and older persons underlined that, if a robot were able to complete some tasks generally performed by caregivers, this might effectively optimize available care resources. Specifically, Pepper can potentially improve caregiving by doing certain tasks for the caregivers, as nursing care staff would not be available to talk for the whole day. As one elder participant put it: “They [the residents) cannot have one nurse who would talk with them the whole day” (Older person 9). One participant also said that using personalized Pepper robots would have been beneficial since they could talk in her voice, a feature which have de-escalated the situation her mother was in where the participant felt unable to attend as a family caregiver. Thus, all three participant groups deemed robots as tools that could **reduce caregiving stress** (Fig. 1).

*Professional Caregiver 23:* We had a situation here [at work], … and they [carers of her mother with dementia] called and she [mother] was raging in the background … no one [other family member] was available and I could not leave …. I also understand the urgency of care [of the situation] … and in such a moment, I would be glad for help [to send a Pepper with my voice].

In addition, family caregivers and older persons felt that **caregiving stress can be reduced** and **optimization of care secured** if, for example, sensors were reliably used. Caregivers can determine what the needs of the older persons are, to ensure that they are safe by monitoring them remotely rather than always checking in-person (Fig. 1). Hence, camera and sensors in the nursing home context could help with the shortage of staff members. In a similar vein, the use of these monitoring technologies can help reassure family caregivers that their elder relative is well and that they need not make a visit to confirm that.

*Older person 19:* Well at the moment, I do not see a need for camera. If I were bedridden, things might look different. If it means the nurse does not have to check constantly and can instead see from the camera, “Ah, it’s very urgent, I need to rush,” then I think it would be a good idea. It might also relieve the person a bit.

However, this optimization of resources might reduce interactions between caregivers and care recipients. For example, with the use of ambient sensor technologies, caregivers can rely on these technologies to fulfil some roles that would ordinarily require them to go to the older person’s residence. Professional caregivers and older persons raising this concern were worried that with the cameras, more information is available for the nurses to decide whether a given situation is “worth” an actual visit, potentially resulting in reduced personal contact with the older adult.

*Professional caregiver 9:* Yes. For example, the caregivers can already judge for themselves with cameras whether it is an emergency or not, and maybe they come later or something. But with an emergency alarm, they don’t know if it’s really okay or if they need to come. … I find these two things (sensors in the house and cameras) a bit ethically problematic, even though they agree. Because maybe they need help, but the caregiver decides if they need help or not, or if it is urgent or not. I find that somehow... you do not know how urgent it is from the outside, I think.

### Worries Associated with Technology Use for Older End-users

#### Stigma

Stigma was reported as a worry by very few participants (from the older person and family caregiver groups) when using the wearables only (Fig. 1). The concern was that wearing the device might signal the presence of a care need or deficiency on the part of the user, particularly when the wearable was overtly noticeable. Referring to the use of smartwatches, and particularly to the idea that information or notifications from it may come at odd times, a family caregiver suggested that vibrations are discrete, ensuring that unnecessary attention is not attracted.

*Older person 18:* It [smartwatch] was just so clunky. One immediately sees [and thinks], ah yes, this is an old person who needs a watch like this now. But today I think the design has improved.

*Family caregiver 5:* Well, I think vibration is interesting because it is very discreet and not embarrassing when she’s [older person] in the city in a café or with her friends or something.

#### Risk of Physical Harm

Only in relation to the robot Pepper did the older adult participant group relay concerns regarding potential physical harm (Fig. 1). That is, using the robot could be dangerous if it gets out of control and physically harms the user. The scenarios in which this threat was described were dramatic. Hence, as relayed in the first theme, the participants felt that it was important that the user has control over the technology and can set boundaries for what the technology can and cannot do in order to be safe for older adults.

*Older person 16:* What do I do if the technology isn’t right and it comes and does something, not what I don’t want but what frightens me, what hurts me or something and I can’t stop it. … Yes, yes. Just when he comes and instead of saying “Hello,” with his little robot hands he takes one and tears the arm off, so just about …yes, yes, simply that something is no longer right in the electronics.

#### Over-Reliance on Technology

All three groups worried that the technology may be employed in ways that could result in relying on them too heavily. For instance, professional caregivers said that information collected from the wearable devices could be used in making an assessment about the health of the older patient, whereby the subjective health information given by the older patient is disregarded or not fully considered. Such circumstances could take away what is important for the older patient; that is, to be **heard and trusted** by their professional caregivers (Fig. 1). However, professional caregivers—specifically in the case of older persons with dementia—felt that the older users might not know how to react to the feedback given by the wearable, such as reminders to take medications. Thus, they raised the point that reminders may not work and falls may happen, cautioning against overdependence on such technologies.

*Professional caregiver 18:* Sometimes it might make things more difficult because I would know if something is not right from what they tell me … It might be harder to take their needs seriously if I have clear facts that contradict them.

*Professional caregiver 17*: Mostly, care involves supervision, especially for medication intake. It is often because people forget, or … or [medication is] taken at the wrong time. Even with reminders, it’s a trade-off—some people might benefit from it, thinking, “Oh yes, I forgot,” while others need someone standing right next to them, saying, “Take your pill now.” There are those who are so overwhelmed that they get distracted and forget. And there are those who just don’t understand what it is and throw it away.

Both older participants and family caregiver groups discussed the **increasing physical dependencies** Pepper may induce in older persons, since some activities of daily living may be done by the robot. An older person making this point noted: “Yes, get me a glass of water or … although it would be good if I got up every now and then and went to get it myself, … if I then have the Pepper, then I'll do it no more” (Older person 26). Underlining how undesirable the situation of a user made passive by their over-reliance on technology might be, a family caregiver stated: “Terrible! So no, then one is supported by technology for everything and then would not [need to] take care of oneself anymore?” (Family caregiver 4).

#### Constant Surveillance and Data Concerns

Continuous monitoring was reported by all participant groups as a key concern with the use of wearables and sensor technologies at home. At the same time, this concern did not arise regarding robotic technologies (Fig. 1). For the ambient sensor technologies, older participants felt that certain parts of a home are more private such that they might be less comfortable with the presence of sensors there: “ … So I mean camera, let’s say at the entrance, I would also think it’s something good. But camera on every corner, I don’t know if you’ll be a little worried, if you know [it’s there]” (Older person 5). As third parties who are subject to such continuous monitoring, the professional caregivers reported feeling mistrusted and assessed.

*Professional caregiver 15:* … Especially when you are really being checked to see if you’re really where you’re supposed to be at that time, right. I just have to say … No, very unpleasant … Even though I did not do anything wrong. It is like that sometimes.

A few participants said that data protection is not something that they think about, indicating that whosoever wants the data can have them. Others were sceptical of sharing data with insurance and banks: “Suddenly the health insurance company somehow notices, oh, they never move, then I increase the premium there a little or with someone else, they do something crazy, and then there too, so that’s the way it is” (Family caregiver 6).

### General Worries about Technology Use in Elder Care

#### Fear of Replacing Human Care

The concern that less human care will be given to older persons as a result of the use of sensor cameras was evident from the above section on the optimization of care resources. However, we found that this fear of the replacement of human care was expressed more heavily in the case of the robot Pepper by the three participant groups (Fig. 1). They reported that having or seeing a Pepper feels like it is already replacing people a little bit, and that was not acceptable because the nursing profession is about building interpersonal relationships. Although there was openness to using Pepper for supportive purposes (i.e. helping with practical activities), it was underlined that it should not become a norm. A participant stated: “It would be a nightmare to have a nursing assistant sitting in an office and giving orders to five robots outside to provide care” (Professional caregiver 22).

Many older persons reported that they cannot imagine using robots for care because the human aspect would be lost, which to them was very important. One participant underlining the little human contact received in nursing care stated that he still values the human contact he gets from the people bringing food, as they get to know each other after a while and that is good.

*Older person 22:* I am … well, personally I’m just not a fan of any of this stuff [Pepper]. Firstly, this breaks the connection with a person [and it] simply doesn’t just interrupt [human care], but eliminates it. There is even less [human] contact.

#### Deception

Deception was a concern that also arose in the context of the robot Pepper, particularly when talking about personalizing Pepper such that it would have a voice of a loved one (Fig. 1). For instance, one older participant stated: “That would deceive me, I don’t want that. If it’s the son, then it’s the son, then I can react to that” (Older person 23*).*

Professional caregivers noted that they find it bothersome that Pepper “simulates something so human, which it is really not” (Professional caregiver 1). They further added that it may be terrible, and at times distressing, that the robot could mimic a person that the user may not know. The emphasis they placed on what is true was clear, and they were concerned with the potential for mistrust between the older resident and the professional caregiver. This line of argument was revealed by family caregivers and older persons as well. That is, participants noted that the robot should be itself, including its funny manner of speaking, and insisted that it should not copy a family member. It was also said that mimicking family members is in some ways scary, while one participant stressed that he would not want his older father to have the feeling that Pepper was his son.

*Professional caregiver 6:* So intuitively terrible. … So the client in question does not have advanced dementia, I think she would know that [Pepper is not a real person]. But now with someone who has more advanced dementia, that would be a bit of a, yes, fooling.

*Family caregiver 10:* No, I don’t think so. I don’t think so, and I feel now that it [Pepper] should be itself, it shouldn't be a family member. No. I think it’s funny when it talks, but I think Pepper should be Pepper and not somebody else, in my opinion. (Moderator: Ok, can you elaborate a bit more?) Yes, because, from my perspective, I don’t want to give it my personality, I don’t want my father to feel that it’s [me].

## Discussion

Like many studies, several benefits of using new technologies as well as ethical concerns were on the minds of our study participants. The former consisted of benefits such as supporting older end-users with their health and safety management and the optimization of care resources for the caregivers of older persons. The latter included respecting the autonomy of the end user, privacy, deception, and the replacement of human care (Birchley et al. 2017; Coghlan et al. 2021; Marikyan, Papagiannidis, and Alamanos 2019; Novitzky et al. 2015; Sharkey and Sharkey 2012; Sorell and Draper 2014; Vandemeulebroucke et al. 2020; Wilson, Hargreaves, and Hauxwell-Baldwin 2015, 2017). More recently, a systematic review of 156 studies by Felber et al. (2023) mapped the ethical concerns raised when new technologies are used in elder care into the following categories: privacy, autonomy, responsibility, human vs. artificial interactions, trust, ageism, and stigma. These much-discussed ethical concerns may impact the acceptance and long-term use of these technologies for caregiving purposes. However, given that the demand for caregiving is expected to increase as a consequence of the growing number of older persons, limited human resources, and significant technological developments, the use of new technologies in this domain will become more frequent over time. Such situations call for ethical preparedness and ethical sensitivity to recognize and address the ethical blind spots we have teased out from the findings of our qualitative data.

**Fig. 2:**
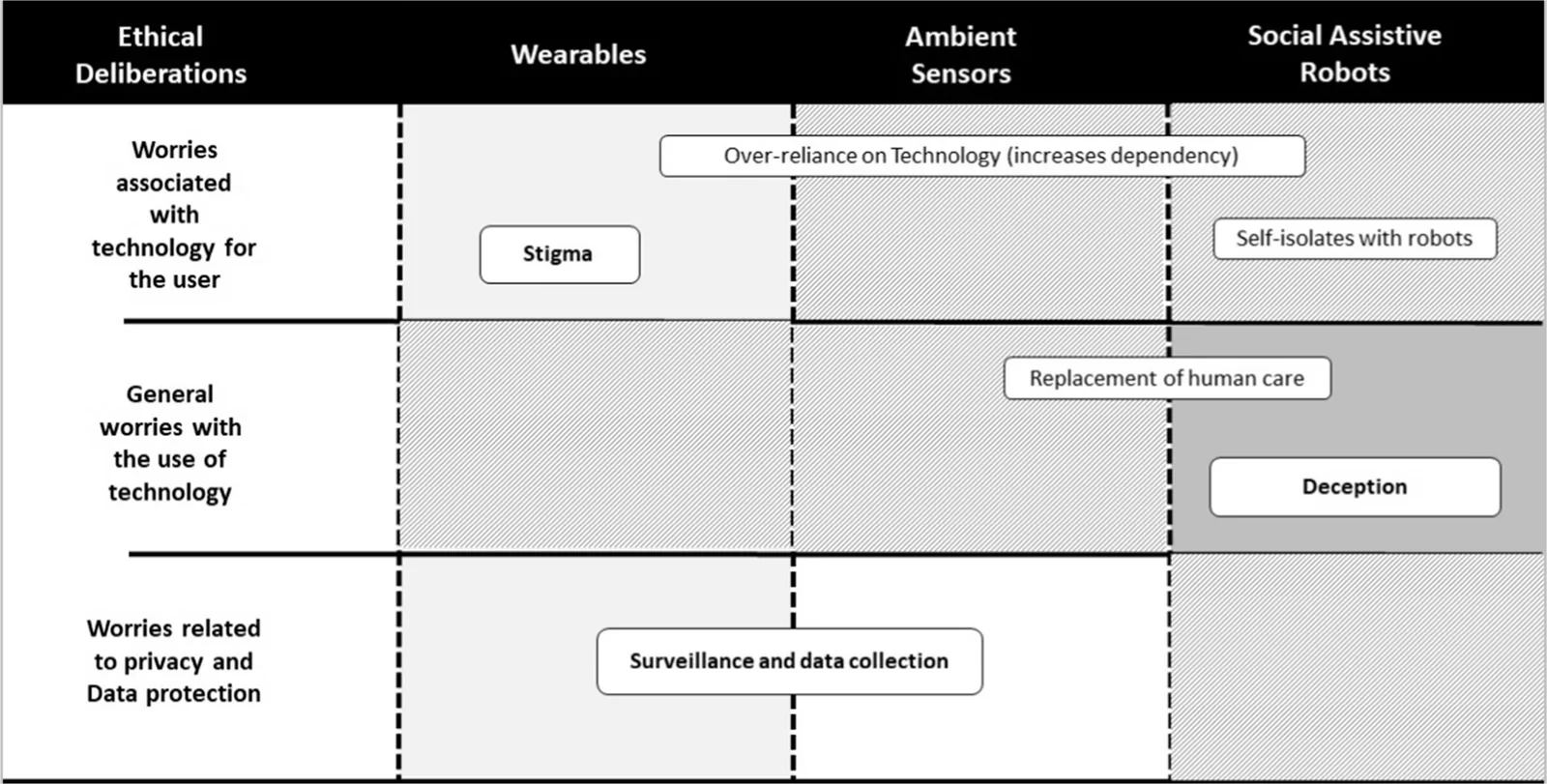
Ethical blind spots and new technologies for elder care

Differing from previous studies on this topic, we show that the importance of ethical concerns is relative to the technology and to participant group. That is, participants undervalued a few ethical issues based on these factors, leading us to the notion of ethical blind spots. Specifically, the issue of stigma was associated with wearables but not with social assistive robots, deception was associated with social assistive robots but not with wearables and ambient technologies, and surveillance was associated with wearables and ambient technologies but not with social assistive robots (Fig. 2). That the use of new technologies may lead to over-reliance was noted for all three types of technology. The replacement of human care was raised as a concern with ambient monitoring sensors as well as with social robots. Finally, the fear that social robots will lead the end-user to self-isolate is also an expected worry for this type of technology (Wright 2023) but likely would not occur with the use of wearable and sensors at home. Thus, in the next sections we only discuss the ethical concerns categorized as ethical blind spots. We are not claiming that the participants were entirely unaware of these ethical issues but rather that the preferential focus they displayed for ethical issues raised by some technologies over others hints at a possible lack of ethical preparedness and sensitivity in addressing all the potential ethical concerns a new technology may pose. Such blind spots may stem from participants lacking the necessary information to anticipate risks, or because certain technological characteristics more readily captured their imagination and concern. For example, in the literature, the negative impacts of AI infrastructures—such as ecological damage and the exploitation of mineworkers—are massively underrepresented in the ethics discourse concerning AI technologies (Hagendorff 2022).

### Stigma

In our findings, stigma associated with technology use arose only in the context of wearables. When a wearable is overtly-noticeable, it may signal the presence of a care need based on the limitations of the user. The other technologies did not raise the concern of stigmatizing older persons. We are sceptical of the idea that wearables render their users particularly susceptible to negative stereotypes about vulnerable groups. First, as technology improves, such systems will become seamlessly integrated into many human activities and environments (Zhu et al. 2022). Smart textiles are an emerging area of research in which textiles can interact with the environment or the user (Shah et al. 2022). Secondly, wearables, like smartwatches, are used increasingly for different purposes, normalizing the technology. The process of normalizing a social behaviour exerts a strong influence on making that behaviour more acceptable (Mihailov et al. 2021), thus reducing potential stigma. By contrast, other studies with competent older adults have reported potential stigma and shame associated with the use of social robots, with some participants feeling that it was making them useless (Coghlan et al. 2021; Dudek et al. 2021; Felber et al. 2023; Hung et al. 2019; Wangmo et al. 2024). It is not surprising that for the robot Pepper, stigma was not a concern. This is because Pepper is not readily available in the market and is currently an expensive piece of technology. However, in the future, both positive and negative associations are possible about individuals who may use Pepper for their care purposes. This would be dependent on the context of how the robot is used and what that use may mean in said context. Such possible future situations call for proactive ways to reduce these associations.

### Deception

Concerns about deception arose only when discussing the robot Pepper, particularly concerning the suggestion of personalizing it to have the voice of a loved one. Professional caregivers also voiced discomfort that Pepper could simulate something so human. They found it troubling that the robot could imitate a person the user knows, calling it fake and deceptive. Participants emphasized that Pepper should remain true to itself and not imitate family members. While not all forms of inauthenticity are inherently deceptive, robots that mimic the voices of loved ones could lead to indirect deception through emotional manipulation. The scholarly literature has also focused on the deceptive capacity of social robots to impersonate human beings (Coeckelbergh 2015; Sharkey and Sharkey 2021; Sparrow and Sparrow 2006). The worry is that social robots could become similar to human agents and, as a result, users would expect from social robots something that they cannot give, like having certain emotions and responsibilities that they are unable to fulfil (Coeckelbergh 2015). Such deception is morally problematic because it exchanges our real-world life for life in an “existence simulator” (Sparrow and Sparrow 2006).

While this type of deception is alarming and captures our attention, there are other more immediate and practical worries related to deception that are overlooked. Although social robots can mimic human behaviour, their physical appearance is distinctly different, allowing human cognition to recognize that a robot is not actually a human being. Deception based on an overestimation of the functionality of technology is of immediate concern. The integration of Large Language Models, such as ChatGPT, into social robots may be more concerning. Moreover, wearables and ambient technologies can be deceptive as well. These devices often integrate Artificial Intelligence-based voice assistants, which are becoming more advanced (Subhash et al. 2020). The voice assistants commonly used in smartphones and smartwatches are likely to be integrated in smart homes. The risk of deception might be higher in the case of wearables and ambient technologies than robotic technologies because the voice assistant lacks a physical form, making it harder to distinguish between artificial and human agents. Without visual cues, it is easier to create the illusion of dealing with a human.

### Privacy

In relaying the benefits of using new technologies to care for older persons, one professional caregiver acknowledged that these also meant monitoring the older person and thus invading their privacy (Tian, Duong et al. 2024). Similarly, when the choice is between privacy protection and individual safety, it is said that privacy violations are seen as justified (Mittelstadt 2017; Zwijsen et al. 2011). Although privacy is a heavily discussed concern when technologies are employed, the participants were not equally worried about surveillance and data sharing for the technologies studied. It has been reported that the population may not be concerned with privacy when the subject is a specific technology but may be more worried when it is about another technology (McLean 2011). Both older adults and professional caregivers associated privacy issues with visual technologies through which they felt observed. However, privacy remained undiscussed concerning the robotic technologies, an issue which must be carefully evaluated (Tian, Duong, et al. 2024). Robotic technology may currently not be as ubiquitous and affordable as monitoring and other sensor technologies. This does not mean that the situation will remain so. It is likely that future innovation in robotic and surveillance technology will converge.

## Limitations

This analysis is part of a larger project that sought to explore the acceptance of smart home health technologies in the care of older persons. Questions related to ethical concerns were not the only focus of this project. Consequently, there could be ethically-relevant information that we did not capture. Furthermore, study participants could have reported the concerns that they felt must be relayed, and may have chosen not discuss others that they deemed unimportant. Social desirability therefore remains an issue despite the interviewers’ assurances that information shared will remain confidential. Furthermore, a potential limitation of bottom-up approaches in qualitative research is the challenge of distinguishing genuinely ethical concerns from other participant-expressed issues. Participants may identify concerns which are salient to them but are not strictly related to ethical considerations. As a qualitative study detailing data captured from a purposive sample of study participants, we further note that our findings are not generalizable. Specifically, issues related to positive and negative associations with the use of technology are likely to impact individuals, families, and other groups differently based on their specific culture and the context(s) in which they find themselves. We nevertheless underline that, unlike other studies, we did capture the perspective of three stakeholder groups and had a sample size that ensured data saturation for the specific topic examined in this paper.

## Conclusion

Our findings reveal how ethical alertness is reduced for certain types of technologies. This was particularly true concerning the “ethical blind spots” of privacy, stigma, and deception. The normalization of some technologies in everyday lives, e.g. wearables, has the potential to reduce the stigma attached to their use, while the use of other technologies might heighten the sense of stigma for older end-users. New worries may be associated with technology use that are culturally specific, an issue we did not evaluate in this single-country study. Thus, there is a great need to carefully examine how ethical concerns vary by culture and context where new technologies are used to care for older persons. Additionally, technological developments are unlikely to remain stand-alone, meaning that they are likely to converge in the future (e.g. social assisted robots will be equipped with advanced sensors, cameras, and AI to not only fulfil practical roles but also to observe and measure user behaviour, etc.). The potential of new technologies to combine several functions also underlines the necessity to further study each advanced technology and its impact on both care recipients and caregivers. Our results are instrumental for a future-oriented approach in which the field of ethics stays ahead of technological trends, harnessing the potential of new technologies while minimizing harms to their end-users.

## Data Availability

Interview data relevant to this paper can be made available to the readers upon reasonable request made to the corresponding author. Furthermore, several of the interview participants have given consent to share their anonymized data, which are available on Open Access Platform, SwissUBase.

## Footnotes

1 For Pepper: https://www.youtube.com/watch?v=UMWkSQz3aOo&t=3s (shown until 1 min 14 s).

## References

[CR1] Birchley, G., R. Huxtable, M. Murtagh, R. ter Meulen, P. Flach, and R. Gooberman-Hill. 2017. Smart homes, private homes? An empirical study of technology researchers’ perceptions of ethical issues in developing smart-home health technologies. *BMC Medical Ethics* 18(1): 23. 10.1186/s12910-017-0183-z PMC5379767 28376811

[CR2] Bowes, A., A. Dawson, and D. Bell. 2012. Ethical implications of lifestyle monitoring data in ageing research. *Information, Communication & Society* 15(1): 5–22.

[CR3] Burrows, A., D. Coyle, and R. Gooberman-Hill. 2018. Privacy, boundaries and smart homes for health: An ethnographic study. *Health & Place* 50(March): 112–118. 10.1016/j.healthplace.2018.01.006 29428688

[CR4] Coeckelbergh, M. 2015. Artificial agents, good care, and modernity. *Theoretical Medicine and Bioethics* 36(4): 265–277. 10.1007/s11017-015-9331-y 26002636

[CR5] Coghlan, S., J. Waycott, A. Lazar, and B.B. Neves. 2021. Dignity, autonomy, and style of company: Dimensions older adults consider for robot companions. *Proceedings of the ACM on Human-Computer Interaction* 5(CSCW1): 104. 10.1145/3449178 PMC8297987 34308262

[CR6] Demiris, G., and B.K. Hensel. 2008. Technologies for an aging society: A systematic review of “smart home” applications. *Yearbook of Medical Informatics* 17(1): 33–40. 18660873

[CR7] Demiris, G., M. Rantz, M. Aud, et al. 2004. Older adults’ attitudes towards and perceptions of ‘smart home’ technologies: A pilot study. *Medical Informatics and the Internet in Medicine* 29(2): 87–94. 10.1080/14639230410001684387 15370989

[CR8] Dudek, M., S. Baisch, M. Knopf, and T. Kolling. 2021. “THIS ISN'T ME!”: The role of age-related self- and user images for robot acceptance by elders. *International Journal of Social Robotics* 13(6): 1173–1187.

[CR9] Felber, N.A., Y.J. Tian, F. Pageau, B.S. Elger, and T. Wangmo. 2023. Mapping ethical issues in the use of smart home health technologies to care for older persons: A systematic review. *BMC Medical Ethics* 24(1): 24. 10.1186/s12910-023-00898-w PMC10061702 36991423

[CR10] Felber, N., W. Lipworth, Y.A. Tian, V. Duong, and T. Wangmo. 2025. Addressing value tensions in the design of technologies to support older persons (AgeTech) using responsible research and innovation and value sensitive design. *Science and Engineering Ethics* 31: 17. 10.1007/s11948-025-00541-4 PMC12213837 40591031

[CR11] Felber, N.A., E. Mihailov, and T. Wangmo. 2025. Virtual reality as a possible aged care technology—Opportunities and prejudices from older persons and their caregivers in a qualitative study. *Fronters in Virtual Reality* 6: 1502450.

[CR12] Felzmann, H., K. Murphy, D. Casey, and O. Beyan. 2015. Robot-assisted care for elderly with dementia: Is there a potential for genuine end-user empowerment? Paper presented at the Emerging Policy and Ethics of Human Robot Interaction conference, March 2, in Portland, Oregon, USA.

[CR13] Hagendorff, T. 2022. Blind spots in AI ethics. *AI and Ethics* 2(4): 851–867.

[CR14] Hsieh, H., and S.E. Shannon. 2005. Three approaches to qualitative content analysis. *Qualitative Health Research* 15(9): 1277–1288. 10.1177/1049732305276687 16204405

[CR15] Huang, G., and S.A. Oteng. 2023. Gerontechnology for better elderly care and life quality: A systematic literature review. *European Journal of Ageing* 20(1): 27. 10.1007/s10433-023-00776-9 PMC10287881 37347277

[CR16] Hung, L., C. Liu, E. Woldum, et al. 2019. The benefits of and barriers to using a social robot PARO in care settings: a scoping review. *BMC Geriatrics* 19(1): 232. 10.1186/s12877-019-1244-6 PMC6708202 31443636

[CR17] Ienca, M., J. Fabrice, B. Elger, et al. 2017. Intelligent assistive technology for Alzheimer's Disease and other dementias: A dystematic review. *Journal of Alzheimer’s Disease* 56(4): 1301–1340. 10.3233/JAD-161037 28222516

[CR18] Ienca, M., M. Lipps, T. Wangmo, F Jotterand, B. Elger, and R.W. Kressig. 2018. Health professionals’ and researchers’ views on Intelligent Assistive Technology for psychogeriatric care. *Gerontechnology* 17(3): 139–150.

[CR19] Kühne, K., M.A. Jeglinski-Mende, M.H. Fischer, and Y. Zhou. 2022. Social robot—Jack of all trades? *Paladyn, Journal of Behavioral Robotics* 13(1): 1–22.

[CR20] Liu, L., E. Stroulia, I. Nikolaidis, A. Miguel-Cruz, and A Rios Rincon. 2016. Smart homes and home health monitoring technologies for older adults: A systematic review. *International Journal of Medical Informatics* 91(July): 44–59. 10.1016/j.ijmedinf.2016.04.007 27185508

[CR21] Majumder, S., E. Aghayi, M. Noferesti, et al. 2017. Smart homes for elderly healthcare—recent advances and research challenges. *Sensors* 17(11): 2496. 10.3390/s17112496 PMC5712846 29088123

[CR22] Marikyan, D., S. Papagiannidis, and E. Alamanos. 2019. A systematic review of the smart home literature: A user perspective. *Technological Forecasting and Social Change* 138(January): 139–154.

[CR23] Mayring, P. 2000. Qualitative content analysis. *Forum Qualitative Sozialforschung / Forum: Qualitative Social Research* 1(2): 20.

[CR24] McLean, A. 2011. Ethical frontiers of ICT and older users: Cultural, pragmatic and ethical issues. *Ethics and Information Technology* 13(4): 313–326.

[CR25] Mihailov, E., B. Rodríguez López, F. Cova, and I.R. Hannikainen. 2021. How pills undermine skills: Moralization of cognitive enhancement and causal selection. *Consciousness and Cognition* 91(May): 103120. 10.1016/j.concog.2021.103120 33774366

[CR26] Mihailov, E., and T. Wangmo. 2025. Does humanness matter? An ethical evaluation of sharing care work with social robots. *Science and Engineering Ethics* 31(4): 1–18 10.1007/s11948-025-00547-y PMC12304055 40721552

[CR27] Mittelstadt, B. 2017. Ethics of the health-related internet of things: A narrative review. *Ethics and Information Technology* 19(3): 157–175.

[CR28] Novitzky, P., A.F. Smeaton, C. Chen, et al. 2015. A review of contemporary work on the ethics of ambient assisted living technologies for people with dementia. *Science and Engineering Ethics* 21(3): 707–765. 10.1007/s11948-014-9552-x 24942810

[CR29] Roesler, E., D. Manzey, and L. Onnasch. 2021. A meta-analysis on the effectiveness of anthropomorphism in human-robot interaction. *Science Robotics* 6(58): eabj5425. 10.1126/scirobotics.abj5425 34516745

[CR30] Schreier, M. 2014. Qualitative content analysis. In *The SAGE Handbook of Qualitative Data Analysis*, edited by U. Flick, 170–182. London: Sage.

[CR31] Shah, M.A., B.M. Pirzada, G. Price, A.L. Shibiru, and A. Qurashi. 2022. Applications of nanotechnology in smart textile industry: A critical review. *Journal of Advanced Research* 38(May): 55–75. 10.1016/j.jare.2022.01.008 PMC9091772 35572402

[CR32] Sharkey, A., and N. Sharkey. 2012. Granny and the robots: Ethical issues in robot care for the elderly. *Ethics and Information Technology* 14(1): 27–40.

[CR33] _____. 2021. We need to talk about deception in social robotics! *Ethics and Information Technology* 23(3): 309–316.

[CR34] Sorell, T., and H. Draper. 2014. Robot carers, ethics, and older people. *Ethics and Information Technology* 16(3): 183–195.

[CR35] Sparrow, R., and L. Sparrow. 2006. In the hands of machines? The future of aged care. *Minds and Machines* 16(2): 141–161.

[CR36] Subhash, S., P.N. Srivatsa, S. Siddesh, A. Ullas, and B. Santhosh. 2020. Artificial intelligence-based voice assistant. Paper presented at the 2020 Fourth World Conference on Smart Trends in Systems, Security and Sustainability (WorldS4), July 27–28 in London, United Kingdom.

[CR37] Tian, Y. J., V. Duong, E. Buhr, N.A. Felber, D.R. Schwab, and T. Wangmo. 2024. Monitored and cared for at home? Privacy concerns when using smart home health technologies to care for older persons. *AJOB Empirical Bioethics* 16(2): 61–76. 10.1080/23294515.2024.2416121 39423332

[CR38] Tian, Y. J., N.A. Felber, F. Pageau, D.R. Schwab, and T. Wangmo. 2024. Benefits and barriers associated with the use of smart home health technologies in the care of older persons: a systematic review. *BMC Geriatrics* 24(1): 152. 10.1186/s12877-024-04702-1 PMC10865618 38355464

[CR39] Turjamaa, R., A. Pehkonen, and M. Kangasniemi, M. 2019. How smart homes are used to support older people: An integrative review. *International Journal of Older People Nursing* 14(4): e12260. 10.1111/opn.12260 31298484

[CR40] Vandemeulebroucke, T., B. Dierckx de Casterlé, L. Welbergen, M. Massart, and C. Gastmans. 2020. The ethics of socially assistive robots in aged care. A focus group study with older adults in Flanders, Belgium. *The Journals of Gerontology: Series B* 75(9): 1996–2007. 10.1093/geronb/gbz070 31131848

[CR41] Vermeer, Y., P. Higgs, and G. Charlesworth. 2019. What do we require from surveillance technology? A review of the needs of people with dementia and informal caregivers. *Journal of Rehabilitation and Assistive Technologies Engineering* 6: 2055668319869517. 10.1177/2055668319869517 PMC6891003 31832230

[CR42] Wangmo, T., V. Duong, N.A. Felber, Y.J. Tian, and E. Mihailov. 2024. No playing around with robots? Ambivalent attitudes toward the use of Paro in elder care. *Nursing Inquiry* 31(3): e12645. 10.1111/nin.12645 38812242

[CR43] Wangmo, T., Y.J. Tian, D. Roulet Schwab, and A.H. Meyer. 2025. Robotic technology in the care of older persons: A cross-sectional national survey among adults in Switzerland, *Innovation in Aging* 9(6): igaf051. 10.1093/geroni/igaf051 PMC12264423 40672553

[CR44] Wilson, C., T. Hargreaves, and R. Hauxwell-Baldwin. 2015. Smart homes and their users: A systematic analysis and key challenges. *Personal and Ubiquitous Computing* 19(2): 463–476.

[CR45] Wilson, C., T. Hargreaves, and R. Hauxwell-Baldwin. 2017. Benefits and risks of smart home technologies. *Energy Policy* 103(April): 72–83.

[CR46] Wright, J. 2023. *Robots won’t save Japan: An ethnography of eldercare automation*. Cornell University Press.

[CR47] Zhu, M., S. Biswas, S.I. Dinulescu, N. Kastor, E.W. Hawkes, and Y. Visell. 2022. Soft, wearable robotics and haptics: Technologies, trends, and emerging applications. *Proceedings of the IEEE* 110(2): 246–272.

[CR48] Zwijsen, S.A., A.R. Niemeijer, and C.M.P.M. Hertogh. 2011.. Ethics of using assistive technology in the care for community-dwelling elderly people: An overview of the literature. *Aging & Mental Health* 15(4): 419–427. 10.1080/13607863.2010.543662 21500008

